# Limitations of pulmonary embolism ICD-10 codes in emergency department administrative data: let the buyer beware

**DOI:** 10.1186/s12874-017-0361-1

**Published:** 2017-06-08

**Authors:** Kristin Burles, Grant Innes, Kevin Senior, Eddy Lang, Andrew McRae

**Affiliations:** 1 Cumming School of Medicine, University of Calgary, Calgary, Alberta Canada; 2 Alberta Health Services, Department of Emergency Medicine, Calgary, Alberta Canada; 3 Emergency Strategic Clinical Network, Alberta Health Services, Calgary, Canada; 4 Emergency Department, C231, Foothills Medical Centre, 1403 29 Street NW, Calgary, Alberta T2N 2T9 Canada

**Keywords:** Pulmonary embolism, PE, ICD-10, Miscoding

## Abstract

**Background:**

Administrative data is a useful tool for research and quality improvement; however, validity of research findings based on these data depends on their reliability. Diagnoses assigned by physicians are subsequently converted by nosologists to ICD-10 codes (International Statistical Classification of Diseases and Related Health Problems, 10th Revision). Several groups have reported ICD-9 coding errors in inpatient data that have implications for research, quality improvement, and policymaking, but few have assessed ICD-10 code validity in ambulatory care databases. Our objective was to evaluate pulmonary embolism (PE) ICD-10 code accuracy in our large, integrated hospital system, and the validity of using these codes for operational and health services research using ED ambulatory care databases.

**Methods:**

Ambulatory care data for patients (age ≥ 18 years) with a PE ICD-10 code (I26.0 and I26.9) were obtained from the records of four urban EDs between July 2013 to January 2015. PE diagnoses were confirmed by reviewing medical records and imaging reports. In cases where chart diagnosis and ICD-10 code were discrepant, chart review was considered correct. Physicians’ written discharge diagnoses were also searched using ‘pulmonary embolism’ and ‘PE’, and patients who were diagnosed with PE but not coded as PE were identified. Coding discrepancies were quantified and described.

**Results:**

One thousand, four hundred and fifty-three ED patients had a PE ICD-10 code. Of these, 257 (17.7%) were false positive, with an incorrectly assigned PE code. Among the 257 false positives, 193 cases had ambiguous ED diagnoses such as ‘rule out PE’ or ‘query PE’, while 64 cases should have had non-PE codes. An additional 117 patients (8.90%) with a PE discharge diagnosis were incorrectly assigned a non-PE ICD-10 code (false negative group). The sensitivity of PE ICD-10 codes in this dataset was 91.1% (95%CI, 89.4–92.6) with a specificity of 99.9% (95%CI, 99.9–99.9). The positive and negative predictive values were 82.3% (95%CI, 80.3–84.2) and 99.9% (95%CI, 99.9–99.9), respectively.

**Conclusions:**

Ambulatory care data, like inpatient data, are subject to coding errors. This confirms the importance of ICD-10 code validation prior to use. The largest proportion of coding errors arises from ambiguous physician documentation; therefore, physicians and data custodians must ensure that quality improvement processes are in place to promote ICD-10 coding accuracy.

## Background

The use of administrative data for research provides multiple advantages: it is readily available, can be used to identify large samples of patients over extended periods, and is relatively inexpensive to acquire. However, the utility of administrative data depends largely on its accuracy and reliability. Administrative database research often relies on diagnostic codes, now defined by the International Statistical Classification of Diseases and Related Health Problems, 10th Revision (ICD-10) [1].

The process for assigning diagnostic codes to patients visiting the emergency department (ED) is standardized in most Canadian hospitals [2]. At the time of patient discharge, ED physicians record a clinical description of the health problems (physician clinical notes) and write a discharge diagnosis or a provisional diagnosis pending further investigations. These discharge diagnoses often fail to conform to ICD definitions, and there is potential for error when nosologists later translate them to ICD codes in hospital administrative databases. Such coding errors may affect research validity, reported disease trends, operational decisions, and health policies.

There is a growing body of research identifying errors associated with ICD-9 diagnostic code assignments in inpatient databases. Yet, few published studies have assessed the accuracy of ICD-10 codes in ED administrative data. Importantly, *O’Malley* et al. report that setting under which ICD codes are assigned is important; so although coding practices are similar for inpatient and ambulatory care settings, each has unique sources of error [3]. Thus, quantification of ICD-10 code accuracy in ED administrative data is important.

Pulmonary embolism (PE) is a potentially life-threatening disease that is diagnostically challenging. With symptoms including dyspnea, chest pain, palpitations, hemoptysis, and or syncope, PE is considered in the differential diagnosis of many cardiopulmonary presentations, thus clinical research to improve PE diagnosis and treatment remains important [4]. Administrative data is a powerful tool for studying PE. The accuracy of diagnostic codes has been well-defined in inpatient data; however, the undifferentiated emergency patient differs from a hospitalized patient, who is more likely to be diagnosed with a PE as a result of their well-recognized increased risk for developing PE. Thus, population differences, in combination with the inherent differences in coding errors within ambulatory and inpatient data, make it difficult to directly compare the validity of ICD codes.

The objective of this study was to assess the validity of PE ICD-10 diagnostic codes as a sole means for identifying diagnoses in ED administrative data.

## Methods

### Data source

Administrative data were obtained from the Ambulatory Care Database of the Alberta Health Services Calgary Zone, located in the province of Alberta, Canada. Given that Alberta has a health care insurance plan that covers all healthcare costs, Alberta Health Services (AHS) is the single health authority in the province and its database includes over 99% of Alberta residents [5]. The Calgary Zone includes four adult acute care hospitals serving a similar demographic population of approximately 1.2 million people, and seeing approximately 325,000 ED visitors yearly. Data extraction included patient age and sex, date and location of hospital visit, presenting complaint, triage note, physician’s written discharge diagnosis, and the subsequently assigned ICD-10 codes. Physician’s clinical notes were also reviewed, providing a narrative and more complete description of the patient’s disposition. Eligible patients were over 18 years of age with an ED visit between July 2013 and January 2015. In Calgary, and across Canada, a coordinator is responsible for establishing and maintaining consistent coding practices for administrative data [2].

### Identification of true positive and false positive PEs

**Fig. 1:**
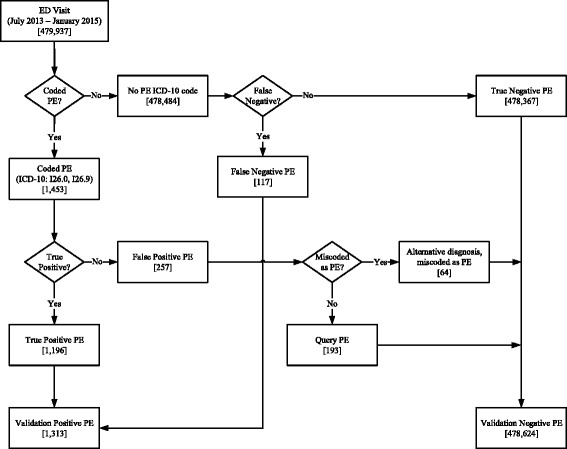
Validation of PE ICD-10 codes. *Coded PE*: patients assigned I26.0 or I26.9 ICD-10 code; *True Positive PE*: patients assigned a PE ICD-10 code whose diagnosis was PE; *False Positive PE*: patients whose chart revealed a diagnosis other than PE; *Miscoded PE*: patients who should have been assigned an alternative code; *Query PE*: patients assigned a PE diagnostic code workup revealed a likely absence of PE; *No PE ICD-10 code:* patients not assigned I26.0 or I26.9 ICD-10 code*; False Negative PE*: patients with a PE who were not assigned a PE ICD-10 code; *True Negative PE*: patients not diagnosed with PE and not assigned a PE ICD-10; *Validation Negative PE*: patients not diagnosed with PE (may or may not be reflected by their ICD-10 code assignment); *Validation Positive PE*: patients diagnosed with PE (may or may not be reflected by their ICD-10 code assignment)

We used ICD-10 codes (I26.9: pulmonary embolism without cor pulmonale, and I26.0: pulmonary embolism with cor pulmonale) to identify patients diagnosed with PE; we refer to these identified patients as the *coded PE* group (Fig. 1). Within the *coded PE* group, we identified true positives by comparing physicians’ clinical notes and written discharge diagnoses to the assigned ICD-10 code. Patients with a congruent PE ICD-10 code were assigned to the *true positive PE* group (Fig. 1). Patients who did not have a PE according to the physicians’ clinical notes and written discharge diagnoses but had a PE ICD-10 code were assigned to the *false positive PE* group. The *false positive PE* group was comprised of two populations: the first had written discharge diagnoses that were obviously non-PE (*miscoded PE* group, Fig. 1). The second group was comprised of discrepant cases where the physician’s written discharge diagnosis was unclear—for example, ‘rule out PE’ or ‘query PE’ (*query PE* group, Fig. 1). Patient medical records and imaging reports were reviewed by two trained investigators to confirm PE diagnosis. For patients whose records indicated a diagnosis other than PE, the most correct diagnosis was documented and the PE ICD-10 code was considered incorrect. An experienced ED physician adjudicated cases of disagreement and complex cases requiring additional expertise.

### Identification of true negative and false negative PEs

Patients with ICD-10 codes for diagnoses other than PE were identified; we refer to these identified patients as the *no PE ICD-10 code* group (Fig. 1). To identify PE cases ‘missed’ by using ICD-10 codes, we performed a free-text search of the physician discharge diagnosis field, looking for the keywords ‘PE’, ‘pulmonary embolism’, ‘pulmonary’, and ‘embolism’. Patients diagnosed with PE without an ICD-10 code for PE were moved to the *false negative* PE group (Fig. 1). The remainder of patients in this study made up the *true negative PE* group (Fig. 1). This group, together with the *false positive PE* group, comprised the *validation negative PE* group (Fig. 1). Similarly, the *true positive PE* and *false negative PE* groups comprised the *validation positive PE* group (Fig. 1).

### Statistical analysis

Four strategies can be used to identify patients diagnosed with PE in administrative data (Table 3). For each strategy, sensitivity (SN), specificity(SP), and positive and negative predictive values (PPV and NPV) with 95% confidence intervals (CIs), were calculated using MedCalc, version 15.11.4 (MedCalc Software, Ostend, Belgium. Accessed *November 29, 2016* at https://www.medcalc.org/calc/diagnostic_test.php). Confidence intervals for sensitivity and specificity are “exact” Clopper-Pearson confidence intervals. Confidence intervals for the predictive values are the standard logit confidence intervals as previously described [6].

## Results

Between July 2013 and January 2015, 479,937 patients visited Calgary EDs, and 1453 (0.30%) received a PE ICD-10 diagnostic code (Fig. 1). However, analysis of the raw data revealed that a subset of patients identified using PE codes were not diagnosed with PE (257 (17.7%)); these errors were preventable, since 64 patients who should have been assigned an alternative code for diagnoses unrelated to PE; for example chest pain, pleural effusion, anxiety, or substance abuse (Table 1). Furthermore, 4 of the 64 *miscoded* patients were randomly assigned a PE ICD-10 code, but their written discharge diagnosis was blank and triage and physician disposition notes indicated no suspicion of PE. The remaining 193 patients were misinterpreted as being positive for PE during code abstraction; they had negative PE investigations and their actual diagnosis remained unspecified. A free text search of the database identified an additional 404 *Query PE* cases; however, these cases were more appropriately coded as chest pain or dyspnea. Thus, the other 67.7% of *Query PE* patients received an appropriate ICD-10 diagnostic code. Conversely, we identified 117 patients (8.9%) with PE who were not assigned a PE code during abstraction; but rather an unrelated ICD-10 code (Table 2). Furthermore, 33 patients diagnosed with PE that were assigned no diagnostic codes at all.

**Table 1:** Summary of patients assigned a PE ICD-10 code who should have been assigned an alternative diagnostic code (*Miscoded PEs*)

Diagnosis	Number coded as PE
Abscess	1
Anxiety	4
Bloody diarrhea	1
Bronchitis	1
Cardiac arrest	1
Chest pain	9
Colic	1
Dyspnea NYD	4
Elevated lactate	1
Emphysema	1
Exertional dyspnea	2
Fever NYD	1
General malaise	1
Heart failure	1
Homicidal threats	1
Hyperparathyroidism	1
Hypoxia	5
Influenza	1
Malignancy	1
Metastatic cancer	5
No discharge notes	4
Pancreatic cancer	2
Pleural effusion	5
Pneumonia	2
Recent PE	3
Substance abuse	1
Urinary tract infection	1
Venous thromboembolism	1
Weakness	2
Total	64

**Table 2:** Summary of the *False Negative PE* group: patients with PE who were assigned ICD-10 codes other than PE

ICD-10 code	Description	Number assigned code
Blank	No code assigned	33
A41.9	Sepsis, unspecified	1
B65.9	Schistosomiasis, unspecified	1
C34.9	Malignant neoplasm of bronchus or lung, unspecified	1
D64.9	Anemia, unspecified	1
F41.9	Anxiety disorder, unspecified	1
I20.0	Unstable angina	1
I46.9	Cardiac arrest, unspecified	2
I47.1	Supraventricular tachycardia	1
I48.9	Atrial fibrillation or atrial flutter, unspecified	1
I50.0	Congestive heart failure	3
I80.2	Phlebitis and thrombophlebitis of other deep vessels of lower extremities	5
I80.8	Phlebitis and thrombophlebitis of other sites	1
J06.9	Acute upper respiratory infection, unspecified	1
J18.8	Other pneumonia, organism unspecified	1
J18.9	Pneumonia, unspecified	2
J44.1	Chronic obstructive pulmonary disease with acute exacerbation, unspecified	1
J44.0	Chronic obstructive pulmonary disease with acute lower respiratory infection	1
J45.9	Asthma, unspecified	1
J96.9	Respiratory failure, unspecified	1
J90	Pleural effusion, not elsewhere classified	5
K74.6	Other and unspecified cirrhosis of the liver	1
R06.0	Dyspnea	2
R07.3	Other chest pain	1
R07.4	Chest pain, unspecified	12
R09.0	Asphyxia	3
R42	Dizziness and giddiness	2
R55	Syncope and collapse	4
R57.0	Cardiogenic shock	1
R94.3	Abnormal results of cardiovascular function studies	1
T81.7	Vascular complications following a procedure, not elsewhere classified	20
T84.7	Infection and inflammatory reaction due to other internal orthopedic prosthetic devices, implants, and grafts	1
Z51.2	Other chemotherapy	4
Total		117

We proposed four strategies that could be used to identify PE patients in administrative data. The first utilized PE ICD-10 codes with no further verification (Table 3 – strategy A). *Assumed accuracy* refers to the perception of accuracy held by the investigator who assumes ICD-10 codes are correct; in this situation, SN, SP, PPV, and NPV appeared to be 100.00% (95%CI, 100.00–100.00) (Table 3 – strategy A, *assumed accuracy*). However, we demonstrated that SN and PPV following validation were actually 91.1% (95%CI, 89.4–92.6) and 82.3% (95%CI, 80.3–84.2), respectively (Table 3 – Strategy A, *true accuracy*). The *assumed accuracy* values for strategies B and C were also 100.00% (95%CI, 100.00–100.00) (Table 3 – strategy B, C, *assumed accuracy*); reflecting that without validation, the investigator unknowingly studies PE using inaccurate data. Notably, the true PPV for strategy B, which included a step to identify patients with missed PEs (i.e.: the *false negative PE* group (Fig. 1)), was was 83.6% (95%CI, 81.9–85.2) (Table 3 – Strategy B, *true accuracy*). Conversely, the SN for strategy C, which instead included a step to remove incorrectly coded PEs (i.e.: the *false positive PE* group (Fig. 1)) was 91.1% (95%CI, 89.4–92.6). Finally, the SN, SP, PPV, and NPV for strategy D, which involved complete validation with missed and incorrectly PE patients being re-assigned to the appropriate group, were 100.00% (95%CI, 100.00–100.00) (Table 3 – strategy D).

**Table 3:**
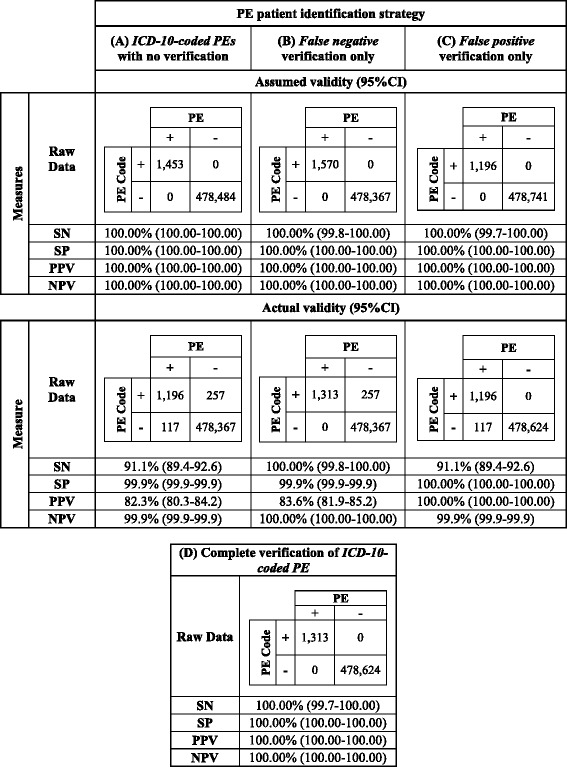
Strategies to identify PE patients

Four strategies can be employed for identifying PE patients in administrative data. Strategy **(A)** uses ICD-10 codes to identify PE patients and employs no verification methods. The *assumed validity* represents how statistical values would appear to an investigator who used our database and assumed correctness of ICD-10 codes. The *actual validity* demonstrates the true statistical analysis of our database, reflecting the coding errors that we identified. The investigator using a strategy lacking ICD-10 code verification would unknowingly miss false positives and negatives in our database. Strategy **(B)** uses ICD-10 codes, with the additional step of identifying the false negative population and moving them to the PE-positive population. The *assumed validity* represents the statistical values when the investigator assumes that the strategy has captured all PEs in the data, and that all patients were correctly assigned a PE ICD-10 code. The *actual validity* demonstrates the true statistical values of the same strategy; an investigator would unknowingly miss false positives in the data set. Strategy **(C)** uses ICD-10 codes, with the additional step of identifying the false positive PE patients and moving them to the PE-negative group. The *assumed validity* represents the statistical values when the investigator assumes that the strategy has removed all patients that were incorrectly assigned a PE diagnostic code; in this case they assume that there are no PE patients who were missed because they are not assigned a PE diagnostic code. The *actual validity* demonstrates the true statistical values of the same strategy; an investigator assuming all patients diagnosed with PE were assigned the appropriate ICD-10 code for PE would unknowingly miss false negatives in the data set. Strategy **(D)** uses ICD-10 codes to identify PE patients, and takes the further steps to identify the false positive and negative populations, moving them to the PE-negative and PE-positive populations, respectively; this strategy ensures that all patients’ true diagnoses are known

*SN* sensitivity, *SP* specificity, *PPV* positive predictive value, *NPV* negative predictive value

## Discussion

ICD-10 codes are widely used in research involving administrative data and are assumed to be an accurate reflection of disease incidence in the population studied, but several groups have identified ICD coding errors as a threat to research validity. Our calculated SN of 91% is congruent with reported sensitivities of PE ICD-9 codes in inpatient and post-operative patient databases (62–92%) [7–10]. Our calculated SN is on the higher end, perhaps reflecting the the expanded list of diagnostic codes in the ICD-10 schedule, meant to improve SN by allowing for more specific diagnostic code assignment. We also identified a *false positive PE* group, meaning that the PPV of PE ICD-10 codes was only 82.3%. *Scarveli* et al. assessed PE ICD-9 codes in inpatient data and similarly calculated a PPV of 80.5% [11]. They reported a false positive rate of 18.5% [11]. *Casez* et al. determined that inpatient PE ICD-10 codes were 89% sensitive for PE, concluding that this is sufficient to use these codes to identify PE patients, although they did not determine the rate of false negatives in their data [12]. Our work builds on the work of *Casez* et al., though we are more conservative in our conclusions, instead encouraging researchers to validate ED administrative data prior to research use.

Our findings suggest that strategies to prevent coding errors are necessary. Diligence during code abstraction and a requirement for imaging confirmation would reduce the number of false positive *miscodes* (64 in our study). Most false positives in our study were in the *query PE* group — the result of ambiguous physician discharge diagnoses. Health information nosologists may add a “Q” prefix before ICD-10 codes to indicate “query” diagnoses or diagnostic uncertainty. Increased use of this prefix may address a large proportion of these errors, as would clear direction to nosologists as to the types of written diagnosis (e.g. “query PE” or “rule out PE”) that would be appropriate to code with a Q prefix. Further utilization of ICD-10 codes for vague or uncertain diagnoses may also help these errors (see Chapter XVIII: Symptoms, signs and abnormal clinical and laboratory findings, not elsewhere classified (R00-R99) [1]).

Previous work has shown that code abstraction depends more on the quality of physician documentation than on the judgment or experience of the nosologist [13, 14]. Because the largest proportion of coding errors appear to result from ambiguous documentation, physicians need to understand the importance of their written diagnosis. Specifically, in cases where a diagnosis is not yet confirmed, physicians should have a standard approach to documentation. For example, a diagnosis of dyspnea NYD or Chest Pain NYD would be preferable to ‘rule out PE’, and would signal nosologists to look for additional confirmation. Periodic departmental audit and feedback of physician diagnostic coding could also identify situations more likely to contribute to ambiguous documentation. For instance, in situations where a physician hands over to a second physician before the results of diagnostic tests are available, the second physician may not return to the chart and modify the first physician’s tentative discharge diagnosis (‘?PE’). This problem could be largely eliminated by a policy that precludes writing in the discharge diagnosis field until investigations are complete.

If implemented, changes to ICD-10 code abstraction will take time; but there will be no pause in use of administrative data. Thus, we presented four strategies that can be used for studying PE using administrative data: A) utilize PE ICD-10 codes alone to identify patients diagnosed with PE, accepting that saving time occurs at the expense of accuracy; B) employ strategies such as keyword identification to identify missed PEs, recognizing the existence of the *false positive* group; C) instead remove *false positive* patients by reviewing patient charts and imaging studies; acknowledging that *false negative* patients might be missed; or D) employ a complete validation strategy as described in this manuscript, which will be more accurate, at the expensive of increased time and resources. Given the small number of patients diagnosed with PE in a relatively large database, SP and NPV were of little use in our study. We suggest that studies requiring accuracy, including those which assesses individual patient characteristics, might benefit from strategy D. On the other hand, strategy A may be more suitable for studies concerned with the number of patients diagnoses rather than individual demographics, such as those monitoring interventions and disease trends.

This study was limited because we did not seek imaging confirmation for all 1453 cases who had a PE ICD-10 codes. Rather, we only reviewed cases where the ICD-10 code was not congruent with the physician’s discharge diagnosis, meaning that we may have missed additional false positive patients. Also, our study reflects the work of physicians and nosologists in one Canadian region. However, agreement with findings of other groups suggests that our findings can likely be applied generally. Like other investigators, we were constrained by the quality of documentation within medical charts, which often lacked more detailed information regarding the physicians’ diagnostic thought process. Though the power of our statistical analyses were limited by the low prevalence of PE in a very large database, our methodology could be applied during validation of other ICD-10 codes. Diagnoses with increased prevalence would benefit from using our strategies for validation.

Depending on the application, the false positive and negative rates seen in our data are a potential threat to validity of PE studies or initiatives that rely on administrative data. We suspect that non-PE ICD-10 codes are not immune to coding errors, and suggest that a validation strategy be employed when using administrative data. Researchers and healthcare administrators should use caution in using ICD-10 codes from ED ambulatory care databases to identify diagnostic groups without verifying the accuracy of ICD-10 coding.

## Conclusions

Our study shows that ambulatory care data, like inpatient data, are subject to coding errors, and confirms the importance of validating ICD-10 diagnostic code accuracy prior to use for research purposes. We demonstrate four strategies for validating ICD-10 codes in administrative data, and these strategies can be applied broadly. The largest proportion of coding errors arises from ambiguous physician documentation; therefore, physicians and data custodians must ensure that quality improvement processes are in place to promote accuracy of ICD-10 diagnostic coding.

## Abbreviations

CI: Confidence interval
ED: Emergency department
ICD-10 codes: International Statistical Classification of Diseases and Related Health Problems, 10th Revision
NPV: Negative predictive value
PE: Pulmonary embolism
PPV: Positive predictive value
SN: Sensitivity
SP: Specificity
